# CAR-NK as a Rapidly Developed and Efficient Immunotherapeutic Strategy against Cancer

**DOI:** 10.3390/cancers15010117

**Published:** 2022-12-24

**Authors:** Marta Włodarczyk, Beata Pyrzynska

**Affiliations:** 1 Department of Biochemistry and Pharmacogenomics, Faculty of Pharmacy, Medical University of Warsaw, Banacha 1B, 02-097 Warsaw, Poland; 2 Centre for Preclinical Research, Medical University of Warsaw, Banacha 1B, 02-097 Warsaw, Poland; 3 Department of Biochemistry, Medical University of Warsaw, Banacha 1, 02-097 Warsaw, Poland

**Keywords:** chimeric antigen receptors, CAR-NK, CAR-T

## Abstract

**Simple Summary:**

New approaches in adoptive immunotherapy using chimeric antigen receptor (CAR)-modified cells have been developing very quickly in recent years, entering into clinical trials and being accepted by health agencies worldwide. Although the classical CAR therapies using genetically engineered T cells (CAR-T) are quite effective in curing resistant and refractory blood disorders, they are less efficient in fighting solid tumors. Therefore, intense research is ongoing to modify the CAR constructs and to use different types of immune cells as platforms for CAR-based therapies in order to make them more efficient and safer. This review summarizes new approaches to CAR therapy, with a particular focus on recent achievements and the benefits of genetic engineering of NK cells.

**Abstract:**

Chimeric antigen receptor (CAR)-modified T cell therapy has been rapidly developing in recent years, ultimately revolutionizing immunotherapeutic strategies and providing significant anti-tumor potency, mainly in treating hematological neoplasms. However, graft-versus-host disease (GVHD) and other adverse effects, such as cytokine release syndromes (CRS) and neurotoxicity associated with CAR-T cell infusion, have raised some concerns about the broad application of this therapy. Natural killer (NK) cells have been identified as promising alternative platforms for CAR-based therapies because of their unique features, such as a lack of human leukocyte antigen (HLA)-matching restriction, superior safety, and better anti-tumor activity when compared with CAR-T cells. The lack of CRS, neurotoxicity, or GVHD, in the case of CAR-NK therapy, in addition to the possibility of using allogeneic NK cells as a CAR platform for “off-the-shelf” therapy, opens new windows for strategic opportunities. This review underlines recent design achievements in CAR constructs and summarizes preclinical studies’ results regarding CAR-NK therapies’ safety and anti-tumor potency. Additionally, new approaches in CAR-NK technology are briefly described, and currently registered clinical trials are listed.

## 1. Introduction

**Figure 1:**
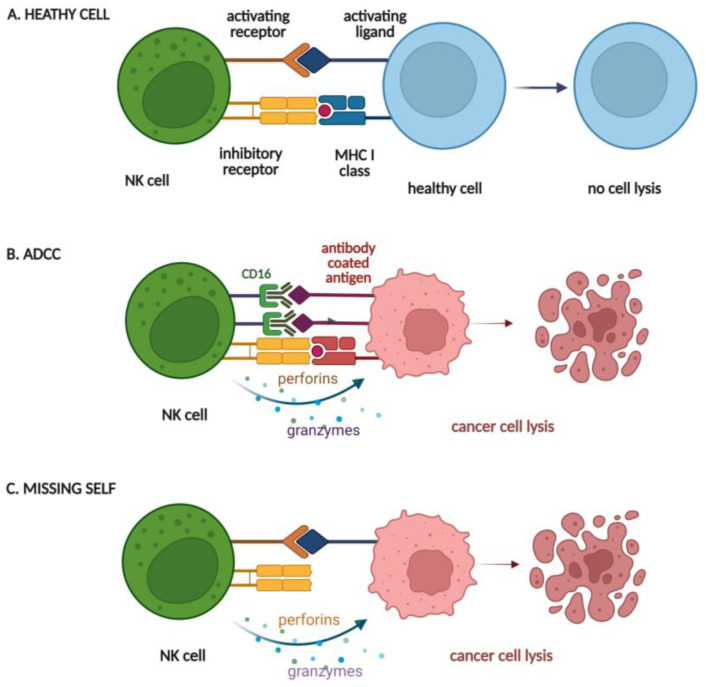
Schematic presenting mechanisms of recognition of healthy cell (**A**), antibody-coated target cell (**B**), or cancer cell missing MHC-I expression (**C**) by NK cell, followed by degranulation of NK cell (**B**,**C**) and cancer cell lysis.

NK cells are large granular lymphocytes which are a component of the innate (non-specific) immune defense system. They mature in the bone marrow, lymph nodes, spleen, thymus, and tonsils, and constitute about 5–10% of circulating lymphocytes in the peripheral blood (reviewed in [1]). NK cells are essential components of the anti-tumor, anti-microbial, and anti-parasite defenses of our immune system. Most NK cells recognize antibody-coated tumor cells via their CD16 receptors (also known as FcγRIIIa), the first step that triggers the activation of antibody-dependent cellular cytotoxicity (ADCC). Unlike T lymphocytes, the action of NK cells is less specific, which causes them to respond rapidly to the presence of transformed or infected cells without a prior need for antigen priming or induction of a specific immune response. However, the activation of NK cells is still strictly controlled by the integration of a variety of signals obtained by the activating and inhibitory receptors, as well as cytokine and chemokine receptors on the surface of NK cells (reviewed in [2]). For example, normal cells express MHC class I (MHC-I) molecules, and their proper amount on the cell surface inhibits NK cell activation via interaction with inhibitory killer immunoglobulin receptors (KIR) on their surface. In contrast, cancer cells synthesize a reduced amount of MHC I molecules, leading to reduced signaling from inhibitory receptors and, eventually, activation of NK cells (schema in Figure 1). 

In the case of both cytotoxic T cells and NK cells, similar molecular mechanisms are responsible for their effector function, including (i) degranulation that relies on the directed release of a membrane-disrupting protein (called perforin) and a family of proteases (called granzymes) from the secretory lysosomes; (ii) production of cytokines, such as tumor necrosis factor (TNF-α) and interferon-gamma (INF-γ), as well as production of membrane proteins, such as Fas ligand (FasL) or TNF-related apoptosis-inducing ligand (TRAIL), which are able to activate the death receptor-mediated killing of target cells [3,4,5].

The efficient cytotoxic activity of T and NK cells makes them the natural choice of cells employed in adoptive cell therapy for cancer, first introduced in the 1980s [6] and later applied in clinical trials to treat metastatic melanoma [7]. Subsequently, AIET therapy (autologous immune enhancement therapy), which uses autologous (patient’s own) NK cells and activated T cells to treat various cancers, was developed by Terunuma et al. [8]. In this technique, the NK cells are obtained from the peripheral blood lymphocytes and processed by a selective immune cell expansion kit without needing feeder cells. In the early nineties of the twentieth century, scientists developed the first engineered T cell with a chimeric T-Cell Receptor (TCR) molecule [9] (reviewed in [10]). In recent years, the genetic engineering of cytotoxic lymphocytes to express membrane receptors, called chimeric antigen receptors (CARs), has become one of the most crucial breakthrough approaches in cancer immunotherapy (reviewed in [11,12]). The recognition of antigens on the surface of tumor cells by CAR is independent of the MHC engagement (in contrast to the recognition by the endogenous TCR). The targeting of tumor-associated antigens induces cellular signaling in CAR-bearing lymphocytes, which drives their proliferation, cytokine production, and degranulation, leading to a cytotoxic attack directed toward tumor cells [13]. CAR therapy is referred to as “living therapy,” as the genetically modified immune cells multiply in the patient’s body and provide long-term anti-cancer memory.

## 2. CAR Engineering

**Figure 2:**
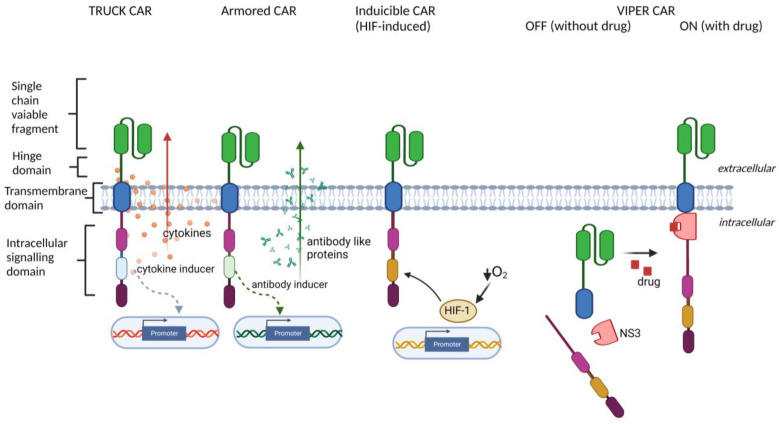
Schema presenting examples of the 4th generation of CAR constructs, including TRUCK CAR, Armored CAR, inducible CAR, and ON/OFF-controllable VIPER CAR.

The term "chimeric" refers to the fact that synthetic CAR molecules are engineered as fusion proteins consisting of at least three essential components; (i) extracellular single chain variable fragment (scFv), classically derived from a murine monoclonal antibody, ensuring specific and efficient recognition of the target antigen on the surface of tumor cell; (ii) extracellular hinge and transmembrane domains; and (iii) intracellular signaling domains ensuring efficient activation of a signaling cascade generating cytotoxic effects toward tumor cells (schema in Figure 2). 

Most often, CAR technology employs the T cells (called CAR-T cells shortly) since they are relatively easy to expand and genetically modify (reviewed in [14]). Briefly, CAR-Ts are made by harvesting T cells from a patient’s blood and reprogramming them in the laboratory to express CAR. Once genetically reprogrammed, grown in large numbers, and administered to the patient’s blood, CAR-T cells circulate through the body and end up binding to a specific surface protein which they recognize. This binding event stimulates immune attack, cancer cell destruction, and further CAR-T cell proliferation, leading to long-term fighting of the tumor cells by CAR-T (reviewed in [15]). The first impressive body of evidence supporting the rationale of such an approach came ten years ago, from studies on genetic engineering and adoptive transfer of T cells expressing CAR proteins that targeted CD19-positive relapsed leukemia [16]. This approach continued to develop further until the first approval of CAR therapy by the US Food and Drug Administration (FDA) in 2017 [17]. After the remarkable success of CAR-T cell therapy for acute lymphoblastic leukemia (ALL), non-Hodgkin lymphoma (NHL), and multiple myeloma, this immunotherapy has been called “The 2018 Advance of the Year” by the American Society of Clinical Oncology (ASCO) [18]. Hundreds of CAR-T cell therapy clinical trials are underway [19].

However, it should be emphasized that CAR-T cell therapies are not free from side effects. For example, some tumor antigens, although highly abundant on the surface of tumor cells, are also present in certain normal cell populations, leading to the recognition and killing of these normal cells by CAR-T cells (a phenomenon called the "on-target/off-tumor" effect). Another possible side effect originates from the uncontrolled proliferation of active CAR-T cells, as well as the excessive release of cytokines and other inflammatory signals throughout the body (called cytokine release syndrome; CRS), which leads to neurotoxicity and other adverse events, ranging from mild fever to life-threatening organ failure (reviewed in [20]). 

Modifications to CAR engineering strategies are the subject of intense research and investigation, aiming to develop a selective and effective approach, with enhanced potency and CAR expression taking place at the tumor site only (reviewed in [21]). 

Recently, the scFv extracellular fragment of CAR constructs has been engineered in various ways by adding the following features:

- Dual or multi-targeting, leading to the recognition of two different epitopes on the same target antigen (to intensify target antigen binding by CAR) or recognition of two or more different antigens (bispecific or multi-specific CARs recognizing antigenic pattern) on the surface of tumor cells (to prevent antigen escape by tumor cells; reviewed in [22]);
- Shorter extracellular fragment, such as single domain variable heavy-chain (VH), derived from camelid antibody (called nanobody; reviewed in [23]) or fully-human heavy-chain-only variable domain (FHVH) instead of conventional scFv fragment (for better expression of smaller CAR constructs on T cells and less immunogenicity induced in the patient’s organism toward foreign human protein [24]);
- switchable CAR-T cells (sCAR-T) with CAR molecules that do not directly recognize tumor antigens, but instead recognize the molecule that targets the antigen, such as the Fab fragment of an antigen-specific recombinant antibody [25,26,27,28] or the adaptor protein zipFv [29], consisting of scFv and a fragment of the leucine zipper, functioning in this case as a “switch” (for more precise control of CAR-T specificity and activity).

The newest, fourth generation of CAR constructs is devoted to improving the tumor penetration and function of CAR-T cells in the immunosuppressive tumor microenvironment (TME), which usually efficiently blocks the function of both endogenous tumor-resident and adoptively transferred T cells, particularly in solid tumors (reviewed in [21,30,31]). The recent innovative approaches to boost CAR-T cell function in TME are shortly summarized below and in Figure 2:

- TRUCK (T cells Redirected for antigen-Unrestricted Cytokine-initiated Killing) approach, based on engineering CAR-T cells to release particular transgenic cytokine upon CAR engagement, including IL-7 [32,33], IL-12 [34,35], IL-15 [36], IL-18 [37,38], IL-23 [39], and IL-33 [40]. TRUCK CARs stimulate the release of cytokines specifically at the tumor site to provide either an auto-stimulatory effect for CAR-bearing cells or activation of other immune cell types in the TME;
- Armored CAR-T cells engineered to express various proteins alongside the CAR (reviewed in [30], such as antibodies or their fragments, which are able to inhibit immune checkpoints [41,42], or dominant-negative TGF-β receptors [43,44], which are able to overcome TGF-β-induced T cell repression in the TME;
- Inducible CAR expression, regulated by specific cellular signaling and transcription factors, including synthetic Notch signaling [45,46], STAT5, AP-1, NFκB [44] (to improve the control over timing and magnitude of CAR expression), or HIF-1α [47] (to restrict the CAR expression to hypoxic areas of the solid tumors);
- ON- and OFF-controllable CAR signaling, regulated by clinically-approved drugs, including CAR regulated by lenalidomide-induced degradation [48], by dasatinib-induced downregulation [49] and by proteolytical cleavage [50,51] (to avoid CAR-T exhaustion and to obtain complete control over CAR activity by drug dosing);
- Multiplex CAR circuits [29,51] combining various CAR technologies, including “AND gate,” SUPRA CAR, universal ON-OFF, and switchboard VIPER CARs (for expanded control over CAR specificity and activation).

The labor-intensive manufacturing and high costs of classical customized CAR-T therapy are associated with the T cells being isolated, modified, and expanded for each patient individually [52]. Therefore, very intensive studies are currently underway to generate Universal CAR-T (UCAR-T) cell therapy (reviewed in [53]) using healthy donor-derived T cells (allogeneic T cells taken from different individuals, immunologically incompatible), which allows for large-scale batch manufacturing and storage with a significantly reduced cost of production. Although such an “off-the-shelf” product can be ready to use when needed, the manufacturing process of UCAR-T requires additional genetic modification, such as deletion of the gene encoding TCR, to prevent severe immune rejection due to the mismatch of MHC between the donor and the patient. 

## 3. Advantages and Limitations of NK Cells as a CAR Platform

**Figure 3:**
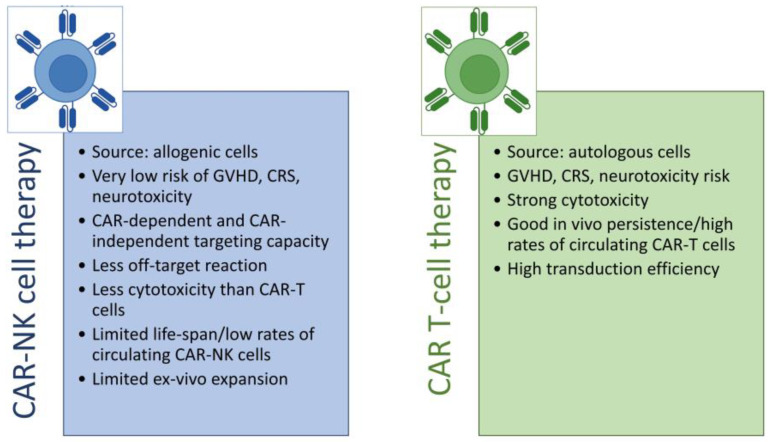
Summary of CAR-NK cell therapy features (list on the left) and CAR-T cell therapy features (list on the right).

The success of novel immunotherapeutic tools, such as CAR-T cells, has inspired scientists to focus on similar genetic modification of other types of cytotoxic lymphocytes, including NK cells (reviewed in [54,55,56]). Additionally, there is a constant demand for exploring other lymphocytes as CAR platforms, since many patients treated with CAR-T still experience a progressive disease [57], severe therapy-related toxicities, or other adverse effects. Most importantly, CAR-NK cells are considered an “off-the-shelf” product, as they can be produced from allogeneic sources, such as the blood of healthy donors. Although NK cells have limited persistence in vivo and are more difficult to employ for genetic modification and expansion in vitro than T cells are, they are considered a safer, more powerful, and universal platform for CAR-based therapies due to their unique biological features (Figure 3) (reviewed in [58]). 

NK cells are quick and potent in eliminating abnormal cells without the prior requirement for antigen priming (in contrast to T cells). The CAR-modified NK cells retain their natural ability to recognize abnormal cells by native receptors in a non-antigen-specific manner and efficiently eliminate them, in addition to CAR-mediated antigen-specific killing.

Importantly, CAR-NK therapies have the potential to be used as “off-the-shelf” types of therapy, since it has been documented that the HLA-mismatched NK cells can be employed as a CAR platform [59]. As a matter of safety, CAR-NK therapy is characterized by less toxicity than CAR-T therapies, with no substantial GVHD or CRS effects [59]. While administration of engineered T cells can cause the release of a variety of cytokines, among them IL-6, which is crucial for the occurrence of CRS (reviewed in [60]), the NK cells produce mainly pro-inflammatory cytokines such as IFN-γ, IL-3, and TNF-α [61]. In the case of allogeneic NK cells’ dose-limiting toxicities, CRS and GvHD are rarely observed, only after being administered in multiple doses or with other agents. For example, in the NSG mouse model, co-expression of CAR and IL-15 in NK cells, when administered in large amounts, resulted in toxicity, indicating the critical need to control NK cell survival in the body [62]. In a recently published paper, expanded autologous NK cells were transduced with NKG2D-based CAR. Such cells exhibited in vitro cytotoxicity against multiple myeloma cells, with minimal activity against normal cells. Mice injected with these CAR-NK cells did not show any sign of GvHD or treatment-related toxicities during the 150 days of the experiment [63]. Infusions of NK cells expressing IL-2, transduced with a lentiviral construct bearing a third-generation CAR which contained CD28 and 4-1BB co-stimulatory molecules, with an Fc fragment inserted between the CD33 scFv and CD28, have been safely used without significant side effects [64]. Cord blood-derived anti-CD19 CAR-NK cells used to cure B-cell malignancies did not produce CRS or symptoms related to neurotoxicity [59]. Additionally, a clinical study of 11 patients with relapsed or refractory CD19-positive tumors showed that most of them responded to CAR-NK treatment without toxic side effects (NCT03056339). CAR-NK therapy is also undoubtedly safer for the treatment of T cell-derived malignancies. Treatment of these tumor types using CAR-T therapy is limited, as both transformed T cells and CAR-T cells (but not CAR-NK cells) share common antigens. Therefore, a high risk of fratricide attack exists, which may lead to the elimination of CAR-T cells themselves. 

The short lifespan of NK cells (typically about two weeks [65]) guarantees the safety of CAR-NK therapy. On the other hand, it compromises the potency and sustained responses to this therapy. Therefore, significant efforts have been made to increase the potency and persistence of CAR-NK treatment without compromising its safety and without reaching NK cell exhaustion. It has been shown that lymphodepletion with fludarabine and cyclophosphamide is crucial to NK cells’ expansion [66]. Additionally, interleukins IL-2 and IL-15 seem to play a critical role in extending the life span and persistence of NK cells in vivo. IL-2 needs to be administrated in vivo to expand the NK cells upon injection. However, high doses of this cytokine are associated with organ toxicity (heart, lung, kidney, and central nervous system), while low doses activate mobilization of suppressive Treg cells, affecting NK cell function [67]. An alternative to IL-2 is the cytokine IL-15, as it does not induce suppressive Tregs and has been shown to improve NK cell cytolytic function [68]. Therefore, CAR constructs incorporating cytokine transgenes could be used for genetic modification of NK cells, allowing parallel expression of CAR and IL-15. Some studies have already proven that such an approach is efficient for improving the persistence of CAR-NK cells [59]. 

Numerous approaches to improving CAR technologies and construct designs have been used to genetically modify NK cells (reviewed in [69]). Unfortunately, NK cells are more resistant to genetic engineering than T cells. The lack of an efficient and standardized method for exogenous gene transfer represents one of the most critical limitations in the production of CAR-NK cells. Retroviral transduction is, so far, the most commonly used method for gene delivery into NK cells (reviewed in [70]). However, it typically ranges between 27–52% of NK cells transduced after a single treatment with retroviruses [71]. The low transduction rates can be improved by stimulating NK cells with IL-2 and IL-21 [72,73]. Lentiviral vectors, despite being a safer method for gene transfer due to reduced insertional mutagenic potential [74], Lentiviral vectors are much less efficient in the modification of NK cells. Constant efforts are made to improve the protocols and achieve at least 15% efficiency [75]. Excellent results (70% efficiency) have, however, been reported upon transduction of primary NK cells with lentiviral vectors which have been pseudotyped with modified envelope proteins derived from baboons or gibbons [76,77,78]. 

Non-viral methods, such as electroporation or lipofection, provide transient expression from the CAR construct, which drops down in 3–5 days since it is usually not integrated into the NK cell genome (reviewed in [70]). Both virus vectors and transfection methods can also be used to deliver the CRISPR/Cas9 genome editing system [79] for precise gene deletion (knockout) [80], repair, or introduction of a new gene (knock-in) [81,82], and could be used for delivery of CAR constructs to NK cells. Recent studies have confirmed the good efficiency of such approaches, including the adeno-associated virus for delivery of the CAR construct and its precise introduction into primary NK cell genome using CRISPR/Cas9 technology [83].

Interesting technologies combining transfection with stable integration of exogenous genes or large DNA fragments into the genome are transposon systems, such as sleeping beauty or PiggyBac [84]. These systems are considered safe and have already been successfully used for “pasting” CAR constructs into the genome of NK cells [85]. Further optimization of this method is needed, since it is still characterized by low efficiency [86]. 

For the generation of CAR-NK therapy, different sources of NK cells can be used, including cord blood, peripheral blood, hematopoietic and induced pluripotent stem cells (iPSCs) [87], and NK-like cell lines (reviewed in [54]). NK cells from different sources exhibit different levels of potency and persistence (reviewed in [88]). However, all of them have already reached clinical trial levels. 

## 4. CAR-Engineered NK-92 Cell Line

The NK-92 cell line derives from the lymphoma cells of a 50-year-old male. It exhibits features of activated NK cells in vitro; its growth is interleukin 2 (IL-2)-dependent, and it expresses NK cell markers, such as the CD56 antigen, activating receptors as well as the NK cell’s typical adhesion molecule CD2. The potent cytotoxicity toward leukemia, lymphoma, myeloma [89], and some solid tumors, as well as the easy expansion of NK-92 in vitro [90], qualified this cell line as a good target for genetic modification with CAR constructs. The high cytotoxicity of NK-92 results from the expression of activating receptors and adhesion molecules, as well as the lack of most KIR inhibitory receptors [91]. NK-92 cells can be easily modified by non-viral transfection methods (reviewed in [92]), and are well-suited for clinical development, since molecularly- and functionally-characterized single-cell clones can be isolated from the NK-92 cell line for further genetic modification with CAR constructs. 

The possibility of continuous expansion in the presence of IL-2, according to the Good Manufacturing Practice (GMP) principles, is the most important advantage of the NK-92 cell line [93]. The safety and clinical activity of NK-92 cells have been initially evaluated in early-phase clinical trials in patients with advanced cancers [94,95]. Positive results encouraged scientists to test the CAR-modified NK-92 in preclinical studies [96,97,98]. However, as in the case of any other tumor cell line that carries a potential tumorigenicity risk, the preparation of the CAR-NK-92 cell line for clinical injection requires the additional step of cell irradiation [99]. Although irradiated NK-92 cells still retain their cytotoxic activity, they have minimal persistence in vivo, typically reaching only one week [100,101]. However, extremely short persistence of NK-92 has also been reported. In the study conducted by Wiliams et al., NK-92 cells were detected in only one of the three patients by flow cytometry 15 minutes after infusion, suggesting a rapid capture of NK-92 from the circulation [102]. Tonn et al. performed Y-chromosome PCR on two female patients who received an NK-92 infusion from a male donor, and also detected a post-infusion signal at very low levels [95]. The lack of an effective diagnostic tool to assess the presence of cells with the CAR transcript [103] can be partially overcome with the direct or indirect labeling of cells with fluorophores, contrast agents, or radioactive isotopes before infusion [104]. However, leakage of tags from cells and release of tags from dying cells is quite common phenomena. A further disadvantage of these solutions is the dilution of the intracellular tracer as the cells divide. Therefore, imaging after a few weeks is not always feasible [105]. Another option is to use a specific label (e.g., antibody or peptide) capable of binding a particular antigen on the surface of target cells in vivo, and followed by optical imaging (OI), magnetic resonance imaging (MR), and nuclear medicine (NM) [105,106]. A disadvantage of this method is the non-specific uptake by healthy tissues (mainly the liver and/or kidneys) due to the physiological biodistribution of the labeling agent.

Interestingly, in patients with colon carcinomas, autologous NK cells labeled with a radiopharmaceutical ^111^In-oxine ex vivo were infused and traced, proving NK cell migration to the tumor metastatic regions [107]. ^111^In-oxine has a 67 h-long half-life; therefore, accumulation of the labeled NK cells can be proven up to 72 h after injection [107]. Recently, Shamalov et al. developed a non-invasive cell tracking technique based on gold nanoparticles to evaluate the kinetics, migration, and biodistribution of NK cells in tumor-bearing mice, which could be applied in the future in human studies [108]. Unfortunately, until now, there has been no precise method for long-term monitoring of CAR-NK therapy persistence in humans.

Compared with the autologous or allogeneic CAR-NK cells and CAR-T therapies, CAR-NK-92 therapy is fairly cheap, since no labor- and cost-intensive cell purification is required in the case of CAR-NK-92 cells. Therefore, even repeated cycles of CAR-modified NK-92 infusions are affordable [109]. The results of clinical trials with NK-92 adoptive therapy have already confirmed the safety of NK-92 cell infusion, even at high doses [93]. A reduced risk of CRS characterizes CAR-engineered NK-92 and NK-92MI (a modified IL-2-independent NK-92 cell line), as they release a small amount of cytokines and exhibit direct killing of tumor cells by releasing toxic granules [110]. 

NK-92 cells are characterized by the lack of CD16 expression on their surface, which makes them unsuitable for ADCC assays. In contrast, an average population of NK cells in peripheral blood exhibits polymorphisms in CD16 receptors, leading to the expression of CD16 proteins with different affinities to Fc fragments of antibodies [111]. Therefore, for CAR applications, some emphasis is placed on genetic modification of NK-92 cells to obtain cell clones with expression of the high-affinity CD16 receptors [112], in order to increase their anti-tumor activity through ADCC (reviewed in [109]). Additionally, some other approaches have been undertaken to obtain clones of NK-92 cells with particular features. Some inhibitory receptors and checkpoint molecules, such as NKG2A, TIGIT, and CBLB, blocking the NK cell activation, can be knocked out by CRISPR/Cas9 gene-editing technology to increase the anti-tumor cytotoxicity of NK-92 cells clones used for modification with CAR constructs [80].

Series of CAR-NK-92 cell lines have been generated and tested in numerous preclinical in vitro and in vivo models of hematologic and solid tumors, proving the good potency of these therapies against different cancer types, including anti-CD3 [113], anti-CD4 [114], anti-CD5 [115], and anti-CD7 [116] CAR-NK-92 treatment for T cell-derived malignancies; anti-EGFR CAR treatment for metastatic breast cancer [117]; and anti-CS1- and anti-CD138 CAR treatment for multiple myeloma [118,119]. Other CAR-modified NK-92 cells used in the preclinical studies targeted the following antigens: CD33 (Rafiq et al., 2015 Cytotherapy [120]), GPA7 [121], HER2 [100,122,123], CD19 (Romanski et al., 2004 Blood [124]), CD20 [125], and GD2 [126]. Additionally, dual and multi-targeting CAR approaches have also been applied to generate CAR-NK-92 cells targeting CD19 and CD138 antigens [127]. Generally, in syngeneic animal models, the treatment with CAR-expressing NK-92 cells results in the activation of endogenous anti-tumor immunity, leading to tumor destruction and the emergence of a long-term immunological memory against tumor rechallenge (reviewed in [110]).

Following CAR-T cell therapy progress, advanced approaches to designing CAR constructs, such as the TRUCK technology, have also been used to modify NK cells in order to improve their anti-tumor function. The work of Rudek et al. investigated the functionality of inducible promoters responsive to NFAT or NFκB transcription factors for the induction of cytokine release. Such TRUCK anti-GD2-CAR-NK-92 cells and -CAR-primary NK cells exhibited great potency, and also enhanced cytotoxic activity and specific activation [128]. 

The first clinical trial results regarding CAR-NK-92 targeting the CD33 antigen on human relapsed and refractory acute myeloid leukemia (AML) cells were reported in 2018 [64]. The paragraph “Clinical trials summary” describes the current clinical trials using CAR-NK-92 therapies. 

## 5. CAR-Engineered Primary NK Cells 

The improvement of GMP-compliant procedures for efficient isolation, ex vivo expansion of blood-derived NK cells, and generation of CAR-NK cells [129] brings hope for allogeneic CAR-NK cell transfer as a potential "off-the-shelf" product that can significantly improve availability and reduce the cost of such therapies. However, the need to use the NK cell isolation kits and either the feeder cells or a variety of cytokines for expansion makes the procedure more expensive compared to the preparation of CAR-NK-92 cells [130]. Umbilical cord blood (UCB) seems to be a better source of NK cells than peripheral blood, since NK cells constitute as much as 30% of circulating lymphocytes. Additionally, UCB is easy to harvest and cryopreserve, which makes UCB-derived NK cells even more suitable for “off-the-shelf” applications [131]. On the other hand, they represent a less mature phenotype, lower tumor cell-directed cytotoxicity, and higher expression of inhibitory receptors, such as NKG2A, when compared to the peripheral blood-derived NK cells [132]. Unfortunately, a small volume of cord blood available from a single donor, makes it necessary to expand isolated NK cells and get the sufficient number for infusion [131]. In contrast to NK-92, the primary NK cells-derived therapy is also difficult to standardize, since the peripheral blood and UCB sources are not homogenous.

Due to the shorter lifespan of NK cells, adoptively transferred NK cells exhibit almost no side effects associated with their use. On the other hand, the limited life span of primary NK cells is a source of short-duration responses to CAR-NK therapy. To overcome this challenge, Liu et al. [62] engineered cord blood-derived NK cells with expression of both CAR construct and interleukin 15 (IL-15), which significantly improved the persistence in vivo as well as the cytotoxic function of NK cells. Such CAR-NK cells, with expanded persistence and equipped with additional expression of inducible caspase 9 as a safety switch in case of NK overactivation, have been successfully used in a small clinical trial for the therapy of relapsed and refractory blood malignancies, and exhibited an excellent safety profile [59]. The GVHD, CRS, neurotoxicity, and elevated levels of inflammatory factors were not found [59]. Current clinical trials using UCB and peripheral blood-derived CAR-NK cells are mentioned below in the paragraph “Clinical trials summary”.

To improve the proliferation and cytotoxic activity of CD33-targeting CAR-NK cells, Albinger et al. [78] demonstrated the possibility of transducing blood-derived primary NK cells with CAR-encoding pseudotyped baboon envelope lentiviral vectors (BaEV-LVs), leading to stable CAR expression in NK cells and their great anti-tumor potency in mouse models of AML [78]. The CD33 antigen is considered an attractive target for therapy to treat myeloid malignancies. Interestingly, Hejazi et al. [133] discovered that the subpopulation of anti-CD33 CAR-NK cells during expansion in vitro undergoes a fratricide-like elimination due to the expression of CD33 antigen on their surface. CD33-positive NK cells exhibit distinctive biological features, such as higher mobilization of cytotoxic granules and higher production of IFNγ and TNFα, while the CD33-negative NK subset shows increased ADCC activity. The expansion of peripheral blood-derived NK cells in NK MACS medium (Miltenyi Biotech), especially, resulted in the most abundant (up to 50%) appearance of the CD33-positive NK cell population [133]. Therefore, depending on the stimulation protocol, it is possible to produce either CD33-positive NK cells that combine effective target cell killing and cytokine production or CD33-negative NK cells that produce fewer cytokines. Still, they are more efficient in antibody-dependent cytotoxicity [133]. 

Although the CAR constructs optimized for T cell signaling, consisting of CD3ζ and 4-1BB, are often used to modify NK cells, several NK-specific signaling domains, such as DAP10, DAP12 [134], and activating receptor 2B4 [97], have been incorporated in CAR constructs (reviewed in [54]). Some new CAR-related approaches are based on employing NKG2D, one of the NK activating receptors (NKGs) with broad target specificity, which induces NK intracellular signaling through DAP10 phosphorylation and can be incorporated in the CAR construct [135]. Recently, such a CAR construct, based on NKG2D, has been tested on genetically-modified NK and T cells. Although the CAR expression upon transduction was found to be more stable in T cells, more significant cytotoxicity toward multiple myeloma was detected in the case of CAR-NK cells [63].

## 6. iPSCs-Derived CAR-NK Cells

As the NK cells derived from either UCB or peripheral blood are neither well-characterized nor homogeneous populations, additional sources of NK cells for modification with CAR constructs have been explored. Some protocols allow the generation of mature NK cells from hematopoietic stem cells (HSCs) [87] or iPSCs [85,136]. NK cells differentiated from HSCs exhibit a mature phenotype, with expression of activating receptors and potent cytotoxicity toward tumor cells [137]. The iPSCs, obtained by reversing the developmental program of somatic cells, are currently an excellent, ever-evolving tool of regenerative medicine [138,139]. Notably, only one CAR-engineered iPSC cell is sufficient to differentiate into many highly homogeneous CAR-NK cells for clinical use. In this case, patients can even be administered with multiple doses of CAR-NK to overcome the limitation of the short lifetime of NK cells. The iPSC-derived NK cells are homogeneous, since they are derived from a clonal population and exhibit long-term expansion potential. CAR-NK therapy with iPSC allows for the large-scale production of a standardized product and its administration in multiple doses [140]. 

Unfortunately, when compared to HSCs, iPSCs are a source of less mature NK cells, with lower expression of CD16 but higher expression of inhibitory receptor NKG2A [141,142]. Nevertheless, in some animal tumor models, the iPSCs-derived CAR-NK cells exhibit similar anti-tumor activity as the peripheral blood-derived CAR-NK cells [141]. Some new improvements in CAR constructs have been tested in iPSCs-derived CAR-NK platforms, such as CAR containing the transmembrane domain of NKG2D and the 2B4 co-stimulatory domain [85], proving their outstanding performance in animal models. In most cases, CAR constructs are introduced into NK cells by retroviral vectors, with their efficiency reaching a maximum of about 50%. Interestingly, Li et al. [85] have proven that iPSCs-derived NK cells can be genetically modified with large DNA fragments by transposon systems, which provide higher biosafety and low immunogenicity compared to viral vectors [143]. Noteworthily, the process of iPSC-NK cell differentiation and maintaining a stable culture of iPSC-NK cells for clinical use remains a challenge, since many factors can influence the iPSC differentiation process: for example, the components of the culture medium [144] and the oxygen supply [145]. The pluripotent stem cells have a propensity for spontaneous differentiation [139]. The development of new protocols allows for the maintenance of a homogenous population of undifferentiated iPSCs characterized by the expression of pluripotent markers, such as stage-specific embryonic antigen 4 (SSEA4) and TRA-1-81. This enables further usage of these cells in a variety of clinical applications [146], including the production of iPSC-derived NK cells. Chen et al. defined Essential 8TM (E8) as a medium designed for the growth and propagation of human PSCs for up to >50 passages [144]. Essential 8TM does not require the presence of BSA (Bovine Serum Albumin) or HSA (Human Serum Albumin), which can cause batch-to-batch variability. The E8 base medium, in the conditions of 5% CO2 availability, provides a pH of 7.4. iPSC cultures require a stable environment of 37 °C, 5% CO2, 5% O2, and around 95% relative humidity. To minimize spontaneous differentiation and chromosomal abnormalities, while inducing cell proliferation, regulation of oxygen concentration to physiological levels (2%–5%) is critical in pluripotent stem cell culture [147,148]. PSCs form colonies, and a suitable material is needed to cover the dish to ensure proper contact between the growing cells, e.g., synthetic extracellular matrix (ECM) [149]. Ni et al. demonstrated that the synthetic peptide-based surface-coating Synthemax II-SC (Corning) could be used in iPSC culture [150]. In addition, vitronectin (VTN), a recombinant ECM xenon-free protein, has been recognized as a component supporting the proliferation rate of human iPSCs under cGMP requirements [151]. Two- and three-dimensional (2D and 3D) culture systems are used to induce hematopoietic differentiation and pass the hematopoietic progenitor cell (HPC) stage to acquire NK cells. Feeder systems assure higher differentiation efficiency, but also increase cost, as more feeder cells are needed for large-scale manufacturing [152].

## 7. CAR-NK as a Therapy for Solid Tumors 

Although quite efficient in curing blood malignancies, CAR therapy faces the following hurdles in curing solid tumors:

- Higher heterogeneity of tumor cells;
- Weak intratumoral penetration and trafficking of CAR-modified immune cells;
- Inhibition of immune cell activation by checkpoint molecules and immunosuppressive TME.

The higher heterogeneity of tumor cells results in a higher probability of target antigen loss on tumor cells during CAR therapy [153]. However, CAR-NK therapy is considered advantageous over CAR-T treatment due to the innate anti-tumor cytolytic capacity of NK cells, which allows the CAR-NK cells to kill tumors using diversified mechanisms, both CAR-dependent and CAR-independent. Additionally, it has been reported that NK cells can eliminate cancer stem cells, usually the most therapy-resistant types of cells residing in solid tumors [154]. The elimination of cancer stem cells is based on decreased MHC-I expression on their surface and the presence of ligands (such as MICA/B proteins) stimulating NKG2D, one of the activating receptors on NK cells [155,156,157]. Certain cytokines secreted by activated NK cells, such as INF-γ and TNF-α, have been reported to stimulate the differentiation of cancer stem cells, leading to the loss of their self-renewal abilities and chemotherapy resistance (reviewed in [158]). 

Several solutions have been implemented to overcome the immunosuppressive TME and enhance the antitumor function of CAR-NK cells. In preclinical studies, some approaches, such as overexpression of chemokine receptors CXCR4 or CXCR1 in CAR-NK cells, have been reported to improve significantly their infiltration into glioblastoma [159] and ovarian cancer [160], respectively. The function of infiltrating immune cells, including NK cells, can, however, be efficiently blocked by tumor cells using the immune checkpoint molecules (reviewed in [161]). Therefore, the blockade of checkpoint receptor TIGIT, associated with the exhaustion of tumor-infiltrating NK cells, has been shown as a promising strategy in the preclinical treatment of colon cancer [162]. An exciting approach to blocking the axis of checkpoint molecules PD1/PD-L1 has been proposed by expression of a chimeric protein PD1-NKG2D-41BB in NK cells, which switches the inhibitory signal from the PD1 receptor to the activating one [163]. 

The cytotoxic functions of T and NK cells can also be efficiently inhibited by various cytokines and metabolites released by both tumor cells and other cell types present in the solid tumor microenvironment (reviewed in [164]). Expressing a dominant-negative TGFβ receptor II, which blocks TGFβ function, in NK cells has been shown as a strategy to overcome the loss of cytotoxicity by NK cells in the glioblastoma TME [165]. 

Numerous tumor-type-associated antigens have been used as targets for CAR-NK therapy in preclinical studies, whereas primarily NK-92 cells have been used as CAR platforms. Progression of high-grade glioblastoma is often driven by the expression of EGFRvIII antigen, a constitutively active mutant of the EGF receptor. Interestingly, to prevent EGFRvIII antigen escape, a therapy with dual-specificity CAR-NK cells, recognizing both mutated and wild-type variants of EGFR, has been shown to prolong the survival of glioblastoma-bearing animals [166,167]. Breast cancers have been efficiently treated by CAR-NK therapy targeting a variety of antigens highly expressed on the surface of breast cancer cells, including HER-2 [100,122,123], tissue factor [168], and EpCAM [169]. Ovarian cancer has been treated by CAR-NK targeting mesothelin antigens [170] or CD24 receptors [171], while pancreatic cancer has been targeted by CAR-NK cells via recognition of ROBO1 antigens [172,173] or folate receptors, used as an antigen for therapy of both tumor types [174,175]. Additionally, c-Met receptors [176] and PSMA [177] have been targeted by CAR-NK cells in therapy for liver and prostate cancers, respectively. An interesting approach with switchable CAR (described in more detail in the “CAR engineering” paragraph), recognizing specific target molecules with GD2 antigen-binding moiety, has been proven useful for the treatment of neuroblastoma and melanoma [126].

## 8. Clinical Trials Summary

In Table 1, we summarize the currently registered clinical studies. According to ClinicalTrials.gov, by November 2022, 42 clinical trials were testing CAR-NK therapy alone or CAR-NK in combination with other drugs. Patient enrollment is ongoing for 27 studies; 4 still need to enter the recruitment phase, and the status of 11 studies is unknown. Most of the presented clinical trials are being conducted in China (n = 28), eight studies are being performed in the United States, one study is located in Germany, and five have an unknown location.

Almost all trials are open-label, single-group assignment studies. Some of them have parallel assignment (NCT05379647 and NCT05487651) or sequential assignment (NCT03692767, NCT05574608, NCT05110742, and NCT05507593). Only one study is characterized as a randomized trial: Phase I/II Study of CAR.70-Engineered IL15-transduced Cord Blood-derived NK Cells in Conjunction With Lymphodepleting Chemotherapy for the Management of Relapse/Refractory Hematological Malignances (NCT05092451).

The clinical trials conducted using CAR-NK have mainly focused on the neoplastic diseases of hematopoietic organs, such as lymphoma and leukemia, NHL, multiple myeloma, and myelodysplastic syndromes (MDS). Some trials concern reproductive tract tumors: prostate cancer, ovarian cancer, testis cancer, endometrial cancer, and epithelial ovarian cancer. Therapy with CAR-NK is also being tested for other solid tumors, such as small cell lung cancer (SCLC), head and neck squamous cell carcinoma (HNSCC), pancreatic cancer, colorectal cancer, and gastroesophageal junction (GEJ) cancer. 

The results from a few clinical trials testing CAR-NK therapy’s potential for solid tumors have been published (reviewed in [178]). The Phase I / II clinical trials of allogeneic cellular immunotherapy with CAR-NK-92 indicate the possibility of treating non-hematological neoplasms with ROBO-1-CAR-NK cells [179,180]. The combination of CAR-NK cells and anti-cancer drugs is a promising type of therapy to alter the immunosuppressive TME and the metastatic capacity of refractory tumors (reviewed in [178]). 

NKG2D is an activated receptor expressed on the surface of human NK cells and some T cells. However, it is absent in healthy tissue. It plays a vital role in innate immunity, as it is involved in recognizing virus-infected cells and killing cancer cells with NK. Five clinical studies developed NKG2D-based CAR-NK therapies to treat myeloid leukemia and solid tumors. The in vitro studies have already demonstrated the anti-tumor action of NKG2D-DAP10-CD3ζ-CAR-NK cells against osteosarcoma cell lines, pancreatic cancer, and breast cancer. A few NKG2D-based CAR therapies are described, with anti-mesothelin-NKG2D-2B4 CARs showing the best results regarding anti-tumor response, proving the potential of 2B4 as the co-stimulatory domain in CAR constructs (reviewed in [181]).

Table 1 presents clinical trial schemas, including CAR-NK cell infusions combined with chemotherapeutics such as cyclophosphamide, fludarabine, and Cytoxan. Phase II trial of PD-L1-CAR-NK cell immunotherapy, in combination with an IL-15 agonist (N-803) and pembrolizumab (NCT04847466), is currently underway among patients with head and neck cancers and gastric cancers in the United States. A German clinical trial is testing the combination therapy of CAR-NK-92 with the anti-PD-1 antibody Ezabenlimab in Patients With Recurrent HER2-positive Glioblastoma (NCT03383978).

A clinical trial with the CAR NK cells targeting the prostate-specific membrane antigen (PSMA) is being carried out in a group of patients with metastatic castration-resistant prostate cancer (NCT03692663). Published results of combined treatment with CAR NK-92 and anti-PD-L1 monoclonal antibody have confirmed its anti-tumor efficacy against prostate cancer in a mouse model [182].

Another phase I/IIa study (NCT05410717) is being carried out in 40 patients with Claudin 6-positive advanced solid tumors. Engineered Claudin 6-targeting CAR is introduced into NK cells isolated from the peripheral blood of patients with advanced ovarian cancer or other cancers expressing Claudin 6. To enhance the killing capability, the CAR-NK cells are genetically engineered to express and secrete IL7/CCL19 and/or scFvs against PD1/CTLA4/Lag3. 

Table 1 also summarizes the target antigens used in current clinical trials employing CAR-NK cells. The CD19 antigen is the most prevalent in engineering CAR-NK and CAR-T cells [92]. Treatments of B cell tumors based on the anti-CD19 CAR-T cell products axicabtagene ciloleucel and tisagenlecleucel were the first to be approved by FDA (reviewed in [183]). The phase 1 and 2 trial results of 11 patients with relapsed or refractory CD19-positive cancers showed a favorable response to CD19-targeting CAR-NK therapy without developing significant toxic effects (NCT03056339 [59]).

Few CAR-NK therapies target AML with anti-CD33, anti-CLL1, and anti-CD123 CARs. The interest in CD33 is based on the fact that about 85–90% of AML cases express the CD33 antigen, whereas, on normal pluripotent hematopoietic stem cells, CD33 expression is absent [184,185]. The clinical trial results using CD33-CAR NK-92 cell infusion indicated that this therapy is safe, but exhibits low anti-leukemia efficacy [64]. Two clinical trials based on anti-CD33 CAR-NK (NCT05215015 and NCT05008575) are currently recruiting AML patients.

Another molecule, highly expressed in AML blasts but not on normal HSC cells, is the family of 12 C lectin domains (CLL1, also known as CLEC12A and MICL) [186]. The in vitro study confirmed that CLL1 CAR-NK cells efficiently eliminate the primary AML cells (Gurney et al., 2021 Blood [187]). Previously, CLL1 and CD33 were successfully used as targets for AML CAR-T cell therapy (Liu et al., 2018 Blood [188]). Please cite the two in number.

The highly expressed molecule in most AML cases is CD123 (Interleukin-3 receptor alpha; IL-3RA) [189]. NK cells expressing CARs targeting CD123 show antigen-specific anti-AML activity in vitro [190]. NK cells expressing a second-generation CAR directed against CD123 exhibit significantly higher cytotoxicity in AML cell lines [191]. In a planned, but not yet underway, clinical trial (NCT05574608), 12 patients with relapsed/refractory AML will receive fludarabine and cyclophosphamide chemotherapy followed by infusion of CD123-CAR-NK cells.

We found two registered clinical trials based on NK cells engineered with anti-CD22 CARs. Several studies have been performed previously with anti-CD22-CAR T-cells, since the expression of CD22, a sialic acid-binding adhesion molecule, is specific to most B-lineage malignancies [57]. Current clinical trial studies of anti-CD22 CAR-NK cells (NCT03692767) and dual anti-CD19/CD22 CAR-NK cells (NCT03824964) are planned against relapsed and refractory B Cell lymphoma. To prevent antigen escape, the CAR-NK cells simultaneously express the CD22-targeting CAR and CD19-specific T cell receptors. Previous in vitro studies have demonstrated that dual CD19/CD22 CAR-NK cells have anti-tumor activity against the ALL cell line [192]. In the murine xenograft model, CD22-CAR/CD19-engager NK cells recognized tumor cells in an antigen-dependent manner, redirected T cells to tumor cells, and caused significant leukemia regression (Szoor et al., 2017 J Immunol [193]).

For the treatment of relapsed or refractory multiple myeloma, clinical trials with CAR-NK cells that express an anti-BCMA (B-cell maturation antigen highly expressed on myeloma cells) construct, together with chemotherapy, are underway (NCT03940833, NCT05008536). The sources of allogeneic NK cells for these therapies are iPSCs and UCB. In a xenograft mouse model, anti-BCMA CAR-NK cells revealed anti-tumor efficiency against multiple myeloma [194]. Roex et al. developed a dual-CAR NK-92 cell line simultaneously targeting CD19 and BCMA, which efficiently recognize and eliminate single- and double-positive target cells, including primary tumor cells [195]. 

In three clinical trials, CAR-NK cells will target ROBO1 antigen on solid tumors. Bi- and tri-specific antibodies, as well as BiCARs, are novel immunotherapy strategies that can engage the immune effector cells so that they eliminate tumor cells by binding simultaneously to both cell types (NK and tumor cells). The forced proximity between these cells causes the release of cytotoxic molecules by effector cells, followed by apoptosis of tumor cells. This method previously showed good anti-cancer activity against several types of non-Hodgkin lymphomas [196,197]. 

T-cell-like NK cells are cytotoxic T-cells that co-express NK receptors such as CD56, CD16, and/or CD57. They are involved in cytotoxic killing, and, like NK cells, are not restricted by MHC recognition [198]. Two studies (NCT04747093 and NCT03882840) are already recruiting patients with B-cell leukemia/lymphoma/acute lymphoblastic leukemia for therapy based on induced T cell-like NK cells.

## 9. Conclusions

Almost a decade has passed since the *Science* magazine named cancer immunotherapy the “Breakthrough of the Year 2013.” Indeed, today, immunology is the most promising field of cancer research, raising great hopes for finding cures for cancer. Clinical approaches, such as CAR therapy or immune checkpoints blockade with antibodies, have revolutionized medicine. In recent years, more attention has been paid to NK cells and their potential usage in adoptive therapy, including CAR therapy, by administering these genetically modified effector cells to patients in order to fight cancer. There has already been significant evidence collected from the results of in vitro studies and animal models for the effective anti-tumor activity of CAR-NK therapies. In parallel, the engineering of CAR constructs is developing rapidly to create "smart" tools to support and improve existing CAR therapies. Numerous clinical trials using blood- or iPSCs-derived NK cells and NK-like cell lines are currently ongoing. However, further large-scale clinical trials are still required to improve the potency of CAR-NK therapy, not only against the most commonly targeted hematological tumor, but also to tackle solid tumors more efficiently.

## Figures and Tables

**Table 1:** The summary of currently registered clinical trials based on CAR-NK therapy.

Title	Trial Number (Number of Participants); Phase	Cancer Type	Treatment; Dosage †	Location
Intracranial Injection of NK-92/5.28.z Cells in Combination With Intravenous Ezabenlimab in Patients With Recurrent HER2-positive Glioblastoma *	NCT03383978 (42); 1	Glioblastoma	CAR-NK-92/5.28.z Doses: 1 × 10^7^–1 × 10^8^ CAR-NK cells Ezabenlimab: 240 mg The standard therapy for glioblastoma (radiotherapy and alkylating chemotherapy) allowed until two weeks and Temozolomide allowed up to 48 h prior to the study	Germany
Clinical Research of Adoptive BCMA CAR-NK Cells on Relapse/Refractory MM +	NCT03940833 (20); 1,2	Multiple Myeloma	BCMA CAR-NK 92 cells	China
CAR-pNK Cell Immunotherapy in CD7 Positive Leukemia and Lymphoma *	NCT02742727 (10); 1,2	Myeloid Leukemia Acute Precursor T-Cell Lymphoblastic Leukemia-Lymphoma T-cell Prolymphocytic Leukemia	anti-CD7 CAR-pNK cells; The allogeneic NK-92 cell line engineered to contain anti-CD7 attached to TCRzeta, CD28 and 4-1BB signaling domains	China
CAR-pNK Cell Immunotherapy for Relapsed/Refractory CD33+ AML+	NCT02944162 (10); 1,2	Myeloid Leukemia Acute	CD33 CAR-NK NK92cells; chimeric antigen receptor NK92 cells transduced with the anti-CD33 vector anti-CD33 CAR-NK (coupled with CD28, CD137 and CD3 zeta signaling domains)	China
NKG2D CAR-NK Cell Therapy in Patients With Relapsed or Refractory Acute Myeloid Leukemia *	NCT05247957 (9); 1	Myeloid Leukemia Relapsed/Refractory Acute	NKG2D ligand-specific umbilical cord blood CAR-NK cells Doses: 2 × 10^6^/kg, 6 × 10^6^/kg, 18 × 10^6^/kg CAR-NK cells, Preconditioning: standard chemotherapy	China
NKG2D-CAR-NK92 Cells Immunotherapy for Solid Tumors *	NCT05528341 (20); 1	Solid Tumors Relapsed/Refractory	NKG2D-CAR-NK92 cells; CAR-NK92 cells targeting NKG2D ligands, Doses: 0.5 × 10^6^, 2 × 10^6^/kg	China
NKG2D CAR-NK Cell Therapy in Patients With Refractory Metastatic Colorectal Cancer *	NCT05213195 (38); 1	Refractory Metastatic Colorectal Cancer	NKG2DL-CAR-NK cells; CAR-NK cells targeting NKG2D ligands,	China
Pilot Study of NKG2D-Ligand Targeted CAR-NK Cells in Patients With Metastatic Solid Tumors +	NCT03415100 (30); 1	Solid Tumors	NKG2DL-CAR-NK cells; CAR-NK cells targeting NKG2D ligands	China
NKX101, Intravenous Allogeneic CAR NK Cells, in Adults With AML or MDS *	NCT04623944 (90); 1	AML, AML Relapsed/Refractory, Adult MDS	NKX101 allogeneic CAR NK cells targeting NKG2D ligands NK cells from haplo-matched related or unrelated donor Doses: 1 × 10^8^ CAR-NK cells (2 × 10^6^/kg for weight < 50 kg), 1.5 × 10^8^ CAR-NK cells (3 × 10^6^/kg for weight < 50 kg) Lymphodepletion: fludarabine/cyclophosphamide or fludarabine/cytarabine (ara-C)	United States
NKX019, Intravenous Allogeneic Chimeric Antigen Receptor Natural Killer Cells (CAR NK), in Adults With B-cell Cancers *	NCT05020678 (60); 1	Lymphoma, Non-Hodgkin B-cell Acute Lymphoblastic Leukemia, Large B-cell Lymphoma	NKX019 allogeneic CAR NK product targeting CD19 on cells. Doses: 3 × 10^8^ NK cells (2 × 10^6^/kg for weight < 50 kg). Lymphodepletion: fludarabine/cyclophosphamide	United States
Anti-CD19 Universal CAR-NK Cells Therapy Combined With HSCT for B Cell Hematologic Malignancies	NCT05570188 (30); 1,2 (Withdrawn -the principal investigator decides to stop)	B-cell Lymphoma B-cell Leukemia	anti-CD19 UCAR-NK cells; Preconditioning: Hematopoietic Stem Cell Transplantation(HSCT) Doses: 5–10 × 10^6^/kg, 1–2 × 10^7^/kg, 2–5 × 10^7^/kg	China
Anti-CD19 CAR-Engineered NK Cells in the Treatment of Relapsed/Refractory Acute Lymphoblastic Leukemia	NCT05563545 (Completed, November 28, 2023) (21); 1	recurrent or refractory CD19 positive acute lymphoblastic leukemia	CAR-NK-CD19 Cells; Doses: 1.0 × 10^7^, 2.0 × 10^7^ and 3.0 × 10^7^ cells /kg, Lymphodepletion: Fludarabine (25–30 mg/kg), Cyclophosphamide (250–300 mg/kg)	China
Anti-CD19 CAR-Engineered NK Cells in the Treatment of Relapsed/Refractory Acute Lymphoblastic Leukemia *	NCT05410041 (21); 1	lymphocyte leukemia, B-cell non-Hodgkin’s lymphoma and chronic B-lymphocyte leukemia	CAR-NK-CD19 Cells; Doses: 1.0 × 10^7^, 2.0 × 10^7^ and 3.0 × 10^7^ cells /kg, Lymphodepletion: Fludarabine (25–30 mg/kg), Cyclophosphamide (250–300 mg/kg)	China
Natural Killer (NK) Cell Therapy for B-Cell Malignancies *	NCT05379647 (24); 1	B-cell Lymphoma B-cell Acute Lymphoblastic Leukemia	QN-019-a (allogeneic CAR-NK cells targeting CD19) as monotherapy or in combination with Rituximab, Lymphodepletion: Cyclophosphamid, Fludarabine, VP-16	China
Study of Anti-CD19 CAR NK Cells in Relapsed and Refractory B Cell Lymphoma +	NCT03690310 (15); 1	Refractory B-Cell Lymphoma	anti-CD19 CAR NK Cells; Doses: 50–600 10^3^/kg	unknown
Clinical Study of HLA Haploidentical CAR-NK Cells Targeting CD19 in the Treatment of Refractory/Relapsed B-cell NHL *	NCT04887012 (25); 1	B-cell Non-Hodgkin Lymphoma	anti-CD19 CAR-NK; lentiviral vector-transduced HLA haploidentical NK cells express anti-CD19 CAR	China
Anti-CD19 CAR NK Cell Therapy for R/R Non-Hodgkin Lymphoma #	NCT04639739 (9); 1	Non-Hodgkin Lymphoma	anti-CD19 CAR-NK; Doses: 2 × 10^6^ /kg, 6 × 10^6^ /kg, 2 × 10^7^/kg Lymphodepletion: Fludarabine (30 mg/m^2^) Cyclophosphamide (500 mg/m^2^)	China
Anti-CD19 CAR-Engineered NK Cells in the Treatment of Relapsed/Refractory B-cell Malignancies *	NCT05410041 NCT03690310 (9); 1	Lymphocytic Leukemia Chronic/Acute Non-Hodgkin Lymphoma	CAR-NK-CD19 Cells; Derived from allogenic NK cells Doses: 1–3 × 10^7^ /KG, Lymphodepletion: Fludarabine (25–30 mg/kg) Cyclophosphamide (250–300 mg/kg)	China
Allogeneic NK T-Cells Expressing CD19 Specific CAR in B-Cell Malignancies (ANCHOR2) *	NCT05487651 (36); 1	Non-Hodgkin Lymphoma, Relapsed, Adult B-cell Lymphoma B-cell Leukemia	KUR-502 - transduced allogeneic natural killer T cells against CD19 (CD19.CAR-aNKT cells) Doses: 1 × 10^7^/m^2^, 3 × 10^7^/m^2^, 1 × 10^8^/m^2^. Body surface area (BSA) capped at 2.4 m^2^. Lymphodepletion: cyclophosphamide (500 mg/m^2^/day) Fludarabine (30 mg/m^2^/day)	United States
Study of Anti-CD19/CD22 CAR NK Cells in Relapsed and Refractory B Cell Lymphoma +	NCT03824964 (10); 1	Relapsed/Refractory B-Cell Lymphoma	Anti-CD19/CD22 CAR NK Cells; Doses: 50–600 10^3^ cells/kg	unknown
Study of Anti-CD22 CAR NK Cells in Relapsed and Refractory B Cell Lymphoma +	NCT03692767 (9); 1	Relapsed /Refractory B-Cell Lymphoma	Anti-CD22 CAR NK Cells Derived from allogenic NK cells Doses: 50–600 10^3^ cells /kg	unknown
Study of Anti-CD33/CLL1 CAR-NK in Acute Myeloid Leukemia *	NCT05215015 (18); 1	Myeloid Leukemia Acute	Anti-CD33/CLL1 CAR-NK Cells; Doses: 2.0 × 10^9^, 3.0 × 10^9^, 3.0 × 10^9^ cells	China
Anti-CD33 CAR NK Cells in the Treatment of Relapsed/Refractory Acute Myeloid Leukemia *	NCT05008575 (27); 1	Leukemia, Myeloid, Acute	anti-CD33 CAR NK cells; Doses: 6 × 10^8^, 12 × 10^8^, 18 × 10^8^ cells/KG Lymphodepletion: Fludarabine (30 mg/m^2^) Cytoxan (300–500 mg/m^2^)	China
Study of Anti-5T4 CAR-NK Cell Therapy in Advanced Solid Tumors *	NCT05194709 (40); 1	Solid Tumors Advanced	Anti-5T4 Oncofetal Trophoblast Glycoprotein (5T4) Conjugated Antibody Redirecting Natural Killer (CAR-NK) Cells Doses: 3.0 × 10^9^, 4.0 × 10^9^, 4.0 × 10^9^ cells	China
Allogenic CD123-CAR-NK Cells in the Treatment of Refractory/Relapsed Acute Myeloid Leukemia #	NCT05574608 (12); 1	Acute Myeloid Leukemia Refractory/ Recurrent	Allogenic CD123-CAR-NK cells; Doses: 1 × 10^6^/kg, 5 × 10^6^/kg, 2 × 10^7^/kg Lymphodepletion: Fludarabine, Cyclophosphamide	unknown
Phase I/II Study of CAR.70- Engineered IL15-transduced Cord Blood-derived NK Cells in Conjunction With Lymphodepleting Chemotherapy for the Management of Relapse/Refractory Hematological Malignances *	NCT05092451 (94); 1,2	Leukemia, Lymphoma, or Multiple Myeloma	CAR.70/IL15-transduced Cord blood NK cells; Lymphodepletion: Cyclophosphamide Fludarabine phosphate	United States
Anti-BCMA CAR-NK Cell Therapy for the Relapsed or Refractory Multiple Myeloma *	NCT05008536 (27); 1	Multiple Myeloma, Refractory	Anti-BCMA CAR-NK, NK Cells from umbilical cord blood, Doses: 1–3 × 10^6^/KG, 3–6 × 10^6^/KG, 0.6–1.2 × 10^7^/KG Lymphodepletion: Fludarabine (30 mg/m^2^), Cytoxan (300–500 mg/m^2^)	China
Cord Blood Derived Anti-CD19 CAR-Engineered NK Cells for B Lymphoid Malignancies *	NCT04796675 (27); 1	Lymphocytic Leukemia Chronic/Acute Non-Hodgkin’s Lymphoma	Cord blood derived NK cells from healthy donor, transduced with a retroviral vector encoding the anti-CD19 CAR and interleukin-15.CAR-NK-CD19 Cells; Doses: 0.01 × 10^7^, 0.1 × 10^7^, 1.0 × 10^7^/kg Lymphodepletion: fludarabine (30 mg/kg) cyclophosphamide (300 mg/kg)	China
Clinical Study of Cord Blood-derived CAR-NK Cells Targeting CD19 in the Treatment of Refractory/Relapsed B-cell NHL *	NCT05472558 (48); 1	B-cell Non-Hodgkin Lymphoma	anti-CD19 CAR-NK; Doses: 2.5 × 10^8^ cells,5 × 10^8^ cells, 1 × 10^9^ cells	China
Phase I/II Study of CD5 CAR Engineered IL15-Transduced Cord Blood-Derived NK Cells in Conjunction With Lymphodepleting Chemotherapy for the Management of Relapsed/Refractory Hematological Malignances #	NCT05110742 (48); 1,2	Hematological Malignancy	CAR.5/IL15-transduced cord blood NK cells; Doses: 1 × 10^7^, 1 × 10^8^, 1 × 10^9^, 1 × 10^10^ cells Lymphodepletion: Cyclophosphamide and Fludarabine or Fludarabine Phosphate	United States
Umbilical & Cord Blood (CB) Derived CAR-Engineered NK Cells for B Lymphoid Malignancies #	NCT03056339 (36); 1,2	B-Lymphoid Malignancies Lymphocytic Leukemia Chronic/Acute Non-Hodgkin Lymphoma	CAR-NK cells CD19-CD28-zeta-2A-iCasp9-IL15-transduced cord blood natural killer (CB-NK) cells Lymphodepletion: Fludarabine Cyclophosphamide (AP1903 in case of graft-versus-host disease (GvHD) or cytokine release syndrome after the NK cell infusion)	United States
Study of Anti-Mesothelin Car NK Cells in Epithelial Ovarian Cancer +	NCT03692637 (30); 1	Epithelial Ovarian Cancer	anti-Mesothelin CAR NK Cells; Doses: 0.5–3 × 10^6^/kg cells	unknown
Study of Anti-PSMA CAR NK Cell (TABP EIC) in Metastatic Castration-Resistant Prostate Cancer *	NCT03692663 (9); 1	Metastatic Castration-resistant Prostate Cancer	TABP EIC (anti-PSMA CAR NK cells) Doses: 0.5, 10, and 30 × 10^6^ CAR NK cells Lymphodepletion: Cyclophosphamide 250 mg/m^2^, fludarabine 25 mg/m^2^	China
CLDN6-CAR-NK Cell Therapy for Advanced Solid Tumors *	NCT05410717 (40); 1,2	Stage IV Ovarian Cancer Testis Cancer, Refractory Endometrial Cancer Recurrent	CAR-NK cells from patients PMBC; (some CAR-NK cells are genetically engineered to express and secret IL7/CCL19 and/or SCFVs against PD1/CTLA4/Lag3)	China
Study of DLL3-CAR-NK Cells in the Treatment of Extensive Stage Small Cell Lung Cancer *	NCT05507593 (18); 1	SCLC, Extensive Stage	DLL3-CAR-NK cells; Doses: 1 × 10^7^, 1 × 10^8^, 1 ×10^9^ DLL3-CAR-NK cells	China
Irradiated PD-L1 CAR-NK Cells Plus Pembrolizumab Plus N-803 for Subjects With Recurrent/Metastatic Gastric or Head and Neck Cancer *	NCT04847466 (55); 2	Gastroesophageal Junction (GEJ) Cancers Advanced HNSCC	Irradiated PD-L1 CAR-NK Cells Plus Pembrolizumab Plus N-803 Doses: 2 × 10^9^ cells Pembrolizumab 400 mg N-803 (15 mcg/kg)	United States
FT576 in Subjects With Multiple Myeloma * (Allogenic CAR NK cells with BCMA expression)	NCT05182073 (168);1	Multiple Myeloma Myeloma	FT576 as monotherapy and in combination with the monoclonal antibody daratumumab FT576: allogeneic natural killer (NK) cells, derived from a clonal, CD38-knockout, iPSC that expresses BCMA, CAR, high-affinity, non-cleavable CD16 (hnCD16), and IL-15/IL-15 receptor fusion protein (IL-15RF). Lymphodepletion: Cyclophosphamide Fludarabine	United States
Induced-T Cell Like NK Cellular Immunotherapy for Cancer Lack of MHC-I +	NCT03882840 (30); 1,2	Anti-cancer Cell Immunotherapy T Cell and NK Cell	Induced-T cell-like NK cells; T-like NK cells (ITNK) from patient’s T cells,	China
Induced-T Cell Like NK Cells for B Cell Malignancies *	NCT04747093 (12); 1,2	B Cell Leukemia B Cell Lymphoma B-cell Acute Lymphoblastic	CAR-ITNK cells; Induced-T cell-like NK cells with chimeric antigen receptor	China
Clinical Research of ROBO1 Specific BiCAR-NK Cells on Patients With Pancreatic Cancer +	NCT03941457 (9); 1,2	Pancreatic Cancer	BiCAR-NK cells- (ROBO1 CAR-NK cells) Derived from allogenic NK cells	China
Clinical Research of ROBO1 Specific BiCAR-NK/T Cells on Patients With Malignant Tumor +	NCT03931720 (20); 1,2	Malignant Tumor	BiCAR-NK/T cells (ROBO1 CAR-NK/T cells) Derived from allogenic NK cells	China
Clinical Research of ROBO1 Specific CAR-NK Cells on Patients With Solid Tumors +	NCT03940820 (20); 1,2	Solid Tumor	ROBO1 CAR-NK cells; Derived from allogenic NK cells	China

* recruiting; # not yet recruiting; + unknown status; † information about pre-condition treatment, type of cells used, and the dosage of CAR-NK cells, if available.
